# Bivariate Genome-Wide Association Analyses of Femoral Neck Bone Geometry and Appendicular Lean Mass

**DOI:** 10.1371/journal.pone.0027325

**Published:** 2011-11-07

**Authors:** Lu Sun, Li-Jun Tan, Shu-Feng Lei, Xiang-Ding Chen, Xi Li, Rong Pan, Fang Yin, Quan-Wei Liu, Xiao-Feng Yan, Christopher J. Papasian, Hong-Wen Deng

**Affiliations:** 1 Laboratory of Molecular and Statistical Genetics, College of Life Sciences, Hunan Normal University, Changsha, China; 2 Center for Bioinformatics and Genomics, Department of Biostatistics, School of Public Health and Tropical Medicine, Tulane University, New Orleans, Louisiana, United States of America; 3 Departments of Orthopedic Surgery and Basic Medical Sciences, School of Medicine, University of Missouri - Kansas City, Kansas City, Missouri, United States of America; 4 Center of Systematic Biomedical Research, Shanghai University of Science and Technology, Shanghai, China; 5 College of Life Sciences and Technology, Beijing Jiao Tong University, Beijing, China; Wayne State University, United States of America

## Abstract

**Objective:**

Femoral neck geometric parameters (FNGPs), such as periosteal diameter (W), cross-sectional area (CSA), cortical thickness (CT), buckling ratio (BR), and section modulus (Z), are highly genetically correlated with body lean mass. However, the specific SNPs/genes shared by these phenotypes are largely unknown.

**Methods:**

To identify the specific SNPs/genes shared between FNGPs and appendicular lean mass (ALM), we performed an initial bivariate genome-wide association study (GWAS) by scanning ∼690,000 SNPs in 1,627 unrelated Han Chinese adults (802 males and 825 females) and a follow-up replicate study in 2,286 unrelated US Caucasians.

**Results:**

We identified 13 interesting SNPs that may be important for both FNGPs and ALM. Two SNPs, *rs681900* located in the *HK2* (hexokinase 2) gene and *rs11859916* in the *UMOD* (uromodulin) gene, were bivariately associated with FNGPs and ALM (*p* = 7.58×10^−6^ for ALM-BR and *p* = 2.93×10^−6^ for ALM-W, respectively). The associations were then replicated in Caucasians, with corresponding *p* values of 0.024 for *rs681900* and 0.047 for *rs11859916*. Meta-analyses yielded combined *p* values of 3.05×10^−6^ and 2.31×10^−6^ for *rs681900* and *rs11859916*, respectively. Our findings are consistent with previous biological studies that implicated *HK2* and *UMOD* in both FNGPs and ALM. Our study also identified a group of 11 contiguous SNPs, which spanned a region of ∼130 kb, were bivariately associated with FNGPs and ALM, with *p* values ranging from 3.06×10^−7^ to 4.60×10^−6^ for ALM-BR. The region contained two neighboring miRNA coding genes, *MIR873* (MicroRNA873) and *MIR876* (MicroRNA876).

**Conclusion:**

Our study implicated *HK2, UMOD, MIR873* and *MIR876*, as pleiotropic genes underlying variation of both FNGPs and ALM, thus suggesting their important functional roles in co-regulating both FNGPs and ALM.

## Introduction

Osteoporosis is a common disease, particularly among the elderly, characterized by decreased bone strength and increased fracture risk [1], [2]. Hip fracture is the most common and serious type of osteoporotic fracture, often producing prolonged or permanent disability, or even death, for some patients [1], [3]. The musculoskeletal system, however, contains both bone and muscle, and these two tissue types are highly interdependent. Bones sustain mechanical loads and provide load points for muscles, and muscles keep bones in place and are responsible for major mechanical loading of bones [4].

Due to the morbidity, mortality, and health care costs associated with osteoporotic fractures, a variety of phenotypic characteristics have been analyzed for their associations with bone strength, and fracture risk. Bone mineral density (BMD) is considered to be an important, but not exclusive, determining factor for bone strength, and is also associated with fracture risk [5], [6]. Bone geometry, independent of BMD, is another important factor, that determines bone strength and is directly associated with osteoporotic fractures [7]. Several recent studies have reported that femoral neck geometric parameters (FNGPs) such as periosteal diameter (W), cross-sectional area (CSA), cortical thickness (CT), buckling ratio (BR), and section modulus (Z), can be used to improve the accuracy of identifying people at high risk of hip fracture [3], [8], [9], [10].

The muscular component of the musculosketal system, as defined by body lean mass, is also closely associated with human health. Low body lean mass is associated with a series of health problems, such as sarcopenia, impaired protein balance, obesity, and osteoporosis [3], [11]. Not surprisingly, body lean mass and FNGPs are closely related phenotypes. It has been demonstrated that bone geometry can serve as a useful index that represents adaptive responses of bone to altered mechanical loading [12]. Body lean mass, in turn, has been shown to contribute to variations of bone geometry at the femoral neck (FN) [13], [14], potentially due to genetic, mechanical, hormonal and nutritional factors. For example, dynamic strains provided by muscle may be an important stimulus of bone adaptation [14]. From the genetic perspective, previous bivariate quantitative genetic analyses have shown that body lean mass was significantly correlated with FNGPs, and that these phenotypic traits might share some common genetic factors [15]. Subsequently, bivariate whole genome linkage analysis reported that several genomic regions, such as 3q12 and 20q13, were linked with both FNGPs and body lean mass [16]. However, the specific SNPs/genes that are shared between these two phenotypes are largely unknown.

Bivariate GWAS is a newly developed effective approach to detect pleiotropic genes for complex traits [17]. To identify the specific pleiotropic SNPs/genes that contribute to both FNGPs and body lean mass, we performed an initial bivariate GWAS in a large Chinese sample, and a follow-up replicate study in Caucasians. We utilized appendicular lean mass (ALM) as our determinant of lean mass, as several studies have suggested that ALM is a better proxy measure of body skeletal muscle mass than total body lean mass for assessing exercise capacity and predicting related diseases [18], [19], [20]. ALM is calculated as the sum of lean mass in the arms and legs.

## Results

The basic characteristics of the study subjects are shown in Table 1. Generally, FNGPs and ALM in men were higher than those in women. All study parameters, except CT in men, were higher in Caucasians than in Chinese subjects.

**Table 1:** Basic characteristics of study subjects.

Traits	Chinese sample			US sample	
	Male (N = 802)	Female (N = 825)		Male (N = 558)	Female (N = 1728)
Age (years)	31.44±11.98	37.44±13.78		50.19±16.03	51.13±13.00
W (cm)	3.54±0.27	3.12±0.33		3.82±0.33	3.30±0.34
CSA (cm^2^)	2.92±0.50	2.25±0.37		3.08±0.59	2.45±0.47
CT (cm)	0.17±0.03	0.14±0.02		0.16±0.03	0.15±0.03
Z (cm^3^)	1.84±0.38	1.26±0.29		2.12±0.49	1.45±0.36
BR	10.97±2.08	11.08±2.76		12.29±2.57	11.46±2.43
ALM (kg)	23.95±3.20	15.75±2.08		29.89±4.85	20.22±3.53

Note: all the values are means ± SD.

Correlation analysis using our phenotypic data have shown that body lean mass was significantly correlated with W, CSA, CT and Z (Table 2), which is generally consistent with previous studies [15], [16].

**Table 2:** Results of bivariate GWAS for ALM and five FNGPs (*p*<1×10^−5^).

Phenotypes pair	SNP	Chr.	Position	Gene	Allele^1^	MAF^2^	MAF^3^	Bivariate *p*	Replication *p*
ALM-W	*rs6789283*	3	21996951	*-*	C/T	0.041	0.034	1.34×10^−7^	0.774
(0.573^**^)^4^	*rs854140*	5	56985279	*-*	G/A	0.462	0.467	3.50×10^−6^	0.825
(0.501^**^)^5^	*rs17037864*	4	160113841	*C4orf45*	A/G	0.364	0.378	3.93×10^−6^	0.865
	*rs2062713*	12	113660935	*-*	T/C	0.289	0.333	7.33×10^−6^	0.592
	***rs681900***	**2**	**74928475**	***HK2***	**G/A**	**0.162**	**0.133**	**7.58**×**10^−6^**	**0.024**
ALM-CSA	*rs4804662*	19	7481408	*-*	G/A	0.231	0.211	4.72×10^−7^	0.216
(0.729^**^)	*rs10928979*	2	127136698	*-*	G/A	0.371	0.433	6.46×10^−7^	0.441
(0.642^**^)	*rs6789283*	3	21996951	*-*	C/T	0.041	0.011	2.50×10^−6^	0.531
	*rs12098712*	10	5601911	*-*	T/C	0.110	0.122	9.56×10^−6^	0.117
ALM-CT	*rs4507747*	8	141288307	*TRAPPC9*	T/C	0.013	0.000	2.32×10^−6^	0.429
(0.524^**^)	*rs10928979*	2	127136698	*-*	G/A	0.371	0.433	3.74×10^−6^	0.766
(0.450^**^)	*rs4804662*	19	7481408	*-*	G/A	0.231	0.211	3.76×10^−6^	0.116
ALM-Z	*rs6789283*	3	21996951	*-*	C/T	0.041	0.011	7.46×10^−8^	0.656
(0.768^**^)	*rs10928979*	2	127136698	*-*	G/A	0.371	0.433	9.58×10^−7^	0.491
(0.679^**^)	*rs2081106*	5	58514356	*PDE4D*	T/A	0.076	0.056	4.91×10^−6^	0.650
	*rs4804662*	19	7481408	*-*	G/A	0.231	0.211	6.19×10^−6^	0.222
	*rs12098712*	10	5601911	*-*	T/A	0.110	0.122	8.72×10^−6^	0.075
ALM-BR	***rs1368998***	**9**	**28869953**	***MIR873***	**T/G**	**0.115**	**0.111**	**3.06×10^−7^**	**0.961**
(-0.095^**^)	***rs12005658***	**9**	**28858243**	***MIR876***	**A/G**	**0.116**	**0.111**	**3.96**×**10^−7^**	**0.976**
(-0.113^**^)	***rs16913782***	**9**	**28866158**	***MIR876***	**T/C**	**0.116**	**0.111**	**4.66**×**10^−7^**	**0.970**
	***rs969715***	**9**	**28810807**	***MIR876***	**C/T**	**0.117**	**0.122**	**5.49**×**10^−7^**	**0.943**
	***rs11998784***	**9**	**28865332**	***MIR876***	**G/A**	**0.116**	**0.111**	**8.12**×**10^−7^**	**0.997**
	***rs1389728***	**9**	**28807957**	***MIR876***	**C/T**	**0.116**	**0.122**	**9.74**×**10^−7^**	**0.569**
	***rs13299777***	**9**	**28929554**	***MIR873***	**C/T**	**0.116**	**0.122**	**9.83**×**10^−7^**	**0.407**
	***rs3849874***	**9**	**28859369**	***MIR876***	**C/G**	**0.116**	**0.111**	**1.13**×**10^−6^**	**0.968**
	***rs16913751***	**9**	**28843125**	***MIR876***	**T/C**	**0.119**	**0.111**	**1.64**×**10^−6^**	**0.939**
	*rs854140*	5	56985279	*-*	G/A	0.462	0.467	2.10×10**^−^** ^6^	0.773
	***rs10491629***	**9**	**28845155**	***MIR876***	**T/G**	**0.117**	**0.116**	**2.91**×**10^−6^**	**0.911**
	***rs11859916***	**16**	**20258732**	***UMOD***	**A/G**	**0.227**	**0.200**	**2.93**×**10^−6^**	**0.047**
	***rs10491633***	**9**	**28933695**	***MIR873***	**T/C**	**0.115**	**0.122**	**4.60**×**10^−6^**	**0.293**
	*rs4804662*	19	7481408	*-*	G/A	0.231	0.211	8.10×10**^−^** ^6^	0.160

Note:

Bold font: the identified 13 SNPs.

1 The first allele represents the minor allele of each locus.

2 Minor allele frequency calculated in our own Chinese subjects.

3 Minor allele frequency reported for Chinese in the public HapMap HCB database.

4 Phenotype correlation for Chinese sample. ** *p*≤0.01.

5 Phenotype correlation for US data. ** *p*≤0.01.

**Figure 1:**
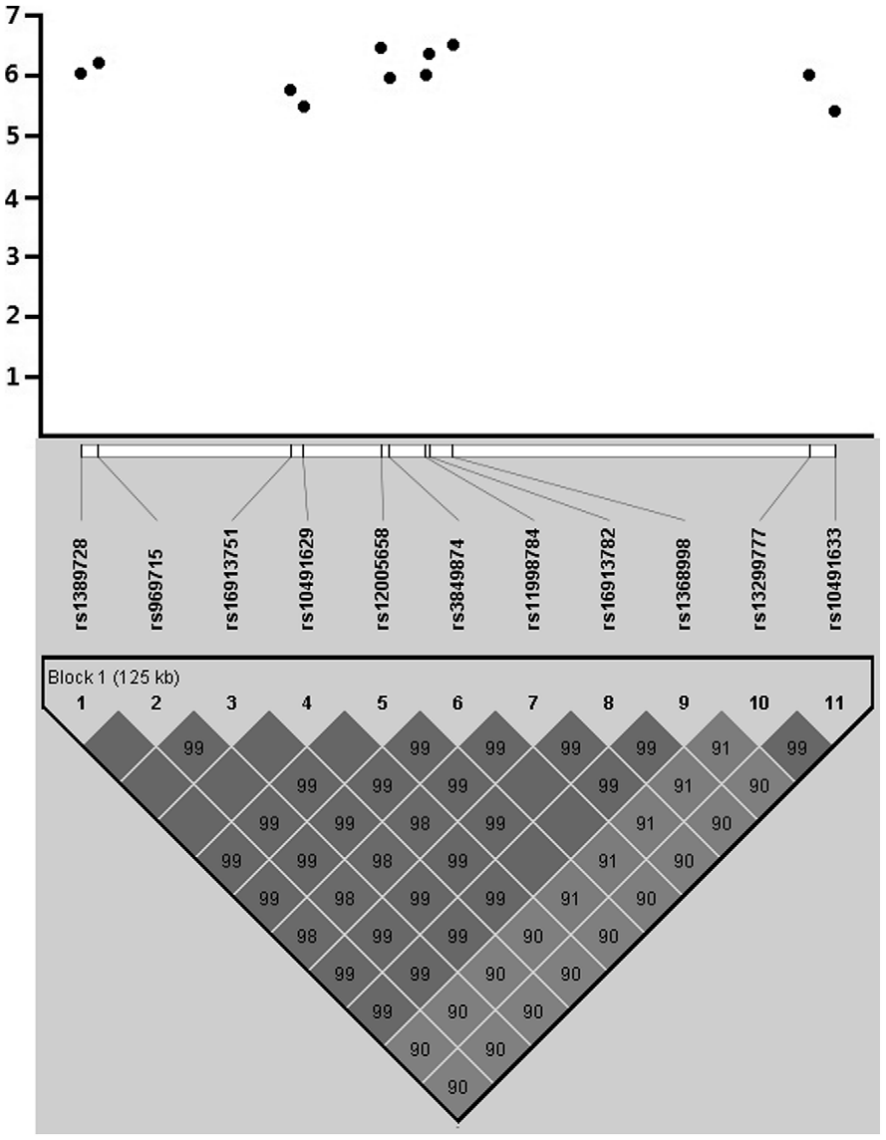
Bivariate associations of 11 contiguous SNPs with ALM-BR in the region of the *MIR876* and *MIR873* genes. Notes: The Y axis is the negative Log10*p* values. The LD between two SNPs is standardized *D'* (*D/D*max).

We identified 13 interesting SNPs that were bivariately associated with ALM and FNGPs. Among them, SNP *rs681900* located in intron 1 of the hexokinase 2 gene (*HK2),* ranked among the top 5 SNPs for bivariate association with ALM-W (*p* = 7.58×10^−6^). Another SNP, *rs11859916* of the uromodulin gene (*UMOD)* ranked among the top 3 loci for associations with ALM-BR (*p* = 2.93×10^−6^). We also found replicate association for these two SNPs in Caucasians, with corresponding *p* values of 0.024 for *rs681900* and 0.047 for *rs11859916* (Table 2). Meta-analyses yielded stronger associations, with pooled *p* values of 3.05×10^−6^ and 2.31×10^−6^ for *rs681900* and *rs11859916*, respectively. A group of 11 contiguous SNPs spanning ∼130 kb region harboring the *MIR876* (MicroRNA876) and *MIR873* (MicroRNA873) genes were strongly associated with ALM-BR, with *p* values ranging from 3.06×10^−7^ to 4.60×10^−6^. Among them, two SNPs, *rs12005658* and *rs3849874*, were located in the promoter region of the *MIR876* gene. As shown in Figure 1, LD signals within this SNP group were very strong and all 11 SNPs were within one haplotype block.

Since the five bone geometry parameters are closely related, we compared the results from the five phenotype pairs for the 13 interesting SNPs to detect common and/or specific SNPs for the five phenotype pairs. As shown in Table 3, three phenotype pairs (ALM-W, ALM-CT, and ALM-BR) generally have stronger association signals than other two (ALM-CSA and ALM-Z) for the 13 interesting SNPs. For SNP rs681900, there were consistently strong association signals across the five phenotype pairs, with p values ranging from 1.57×10^−4^ to 7.58×10^−6^, probably suggesting that this SNP has a common effect on all five phenotype pairs.

**Table 3:** Bivariately/univariate associations for 13 interesting SNPs.

SNP	Role	Bivariate *p* values						univariate *p* values					
		ALM-W	ALM-CSA	ALM-CT	ALM-Z	ALM-BR		ALM	W	CSA	CT	Z	BR
*rs681900*	Intron1	7.58×10^−6^	1.57×10^−4^	5.44×10^−5^	9.39×10^−5^	3.01×10^−5^		0.59	0.06	0.90	0.61	0.95	0.46
*rs11859916*	Intron7	1.58×10^−2^	6.01×10^−2^	3.67×10^−4^	4.12×10^−1^	2.93×10^−6^		0.72	0.03	0.09	0.55	0.03	0.68
*rs12005658*	Promoter	4.60×10^−5^	6.42×10^−3^	4.14×10^−5^	4.61×10^−2^	3.96×10^−7^		0.84	0.87	0.36	0.45	0.76	0.98
*rs16913782*	Upstream	3.37×10^−5^	7.96×10^−3^	6.55×10^−5^	4.18×10^−2^	4.66×10^−7^		0.93	0.87	0.40	0.52	0.80	0.93
*rs969715*	Downstream	3.06×10^−5^	1.97×10^−2^	9.41×10^−5^	8.58×10^−2^	5.49×10^−7^		0.82	0.97	0.64	0.44	0.89	0.78
*rs11998784*	Upstream	9.41×10^−5^	9.25×10^−3^	7.37×10^−5^	6.61×10^−2^	8.12×10^−7^		0.96	1.00	0.37	0.44	0.82	0.87
*rs1389728*	Downstream	4.16×10^−5^	2.31×10^−2^	1.61×10^−4^	8.96×10^−2^	9.74×10^−7^		0.80	0.99	0.63	0.44	0.87	0.79
*rs3849874*	Promoter	8.96×10^−5^	7.99×10^−3^	9.57×10^−5^	4.95×10^−2^	1.13×10^−6^		0.89	0.88	0.39	0.50	0.79	0.96
*rs16913751*	Downstream	1.20×10^−4^	1.07×10^−2^	1.42×10^−4^	6.53×10^−2^	1.64×10^−6^		0.93	0.98	0.55	0.38	0.81	0.76
*rs10491629*	Downstream	1.37×10^−4^	1.04×10^−2^	2.43×10^−4^	4.82×10^−2^	2.91×10^−6^		0.91	0.96	0.57	0.36	0.85	0.73
*rs1368998*	Downstream	3.18×10^−5^	6.29×10^−3^	3.33×10^−5^	4.82×10^−2^	3.06×10^−7^		0.94	0.81	0.28	0.37	0.64	0.96
*rs13299777*	Upstream	8.76×10^−5^	1.51×10^−3^	1.91×10^−5^	3.27×10^−2^	9.83×10^−7^		0.65	0.81	0.93	0.68	0.70	0.71
*rs10491633*	Upstream	1.95×10^−4^	3.21×10^−3^	8.28×10^−5^	4.42×10^−2^	4.60×10^−6^		0.58	0.77	0.79	0.80	0.60	0.76

As shown in Table 3, the bivariate association signals were generally stronger than the univariate association signals, suggesting that bivariate analysis has a higher power to detect shared genetic factors for related phenotype pairs [17]. For example, with SNP rs681900, the univariate associations were not significant for either ALM or W, but bivariate associations were consistently significant for all phenotypic pairs, with p values ranging from 1.57×10^−4^ to 7.58×10^−^6. To provide readers more details about the bivariate GWAS, all the SNPs with *p* value less than 10^−4^ were demonstrated in Appendix S1.

## Discussion

Using the novel multivariate approach, we performed a bivariate GWAS for ALM and FNGPs, and found that 13 interesting SNPs located within or near four genes, *HK2, UMOD, MIR876 and MIR873*, showed strong associations with both femoral neck bone geometry and ALM. The present study represents the first effort to detect shared genetic factors for these closely related phenotypes (ALM and FNGPs).

GWAS provides impressive statistical power for detecting novel genetic variants that underlie common human diseases. To date, however, most published GWAS's utilize a univariate framework to analyze different phenotypes separately. Although these univariate GWA analyses have led to the discovery of novel genes for several complex diseases, this approach often lacks sufficient power to detect pleiotropic genes that may influence multiple phenotypes. Newly developed methodologies for bivariate GWAS's have higher power for detecting pleiotropic genes than univariate approaches [17], [21]. The results shown in Table 3 clearly demonstrate the advantage of bivariate association studies for identifying pleiotropic genes.

The importance of the 13 interesting SNPs is supported by both the statistical evidence provided in this manuscript, and by the known functions of the four genes at the genomic regions containing these 13 interesting SNPs. *HK2* encodes the protein hexokinase 2, the predominant form of hexokinase presented in skeletal muscle. Hexokinase is responsible for phosphorylating glucose to produce glucose-6-phosphate, the first step in most glucose metabolism pathways. Hexokinase 2 is one of the key enzymes involved in regulating glucose metabolism for muscle tissue [22], [23]. Glucose metabolism, one of the most basic cellular biochemical reactions, provides energy and material for fundamental cellular activities such as protein metabolism, cell growth, and proliferation [24], [25], [26], [27]. These activities are essential for normal muscle growth, and may influence lean mass in human [28]. Glucose metabolism is also associated with bone development, as elevated glucose levels have been shown to inhibit calcium uptake and bone mineralization [29]. Moreover, bone resorption is dependent on glucose concentrations. Reduced expression of *HK2* is also associated with non-insulin-dependent diabetes, which may lead to osteoporosis and losses in lean mass [30], [31]. This collective information strongly supports the conclusion that *HK2* is involved in both muscle and bone metabolism.

The *UMOD* gene encodes uromodulin, the most abundant protein in mammalian urine. Uromodulin may influence both bone and muscle by regulating renal excretion of metabolites. Uromodulin is involved in calcium metabolism, and acts as a regulator of calcium oxalate crystallization [32]. Missense mutation of *UMOD* in mice causes moderate increases in plasma calcium concentrations, and significant decreases in bone mineral density and bone mineral content [33]. Uromodulin may also influence lean mass through creatinine, energy, and protein metabolism, as mice with *UMOD* mutations have reduced body weight, including body lean mass, and significant increases in plasma creatinine and urea levels, compared with normal mice [33].

Of particular interest, a group of 11 contiguous SNPs locates within genomic regions containing two miRNA encoding genes (*MIR876* and *MIR873)* were strongly associated with ALM-BR in Chinese. Two of the 11 SNPs, *rs12005658* and *rs3849874* were located in the promoter region of *MIR876*. MicroRNAs (miRNAs) are involved in post-transcriptional regulation of gene expression in multicellular organisms by affecting both the stability and translation of mRNA. We used the TargetScan platform (http://www.targetscan.org release 5.1), web-based software with a low false positive rate, to identify predicted miRNA target genes. *MIR876* encodes two MicroRNAs: miR-876–3p and miR-876–5p. Using TargetScan software, 93 and 148 genes were predicted to be targets for miR-876–3p and miR-876–5p, respectively. Among them, *ACTN4* (actinin, alpha 4) [34], *MBNL1* (muscleblind-like) [35], and *SEPN1* (selenoprotein N, 1) [36] are involved in muscle metabolism, and *ANKH* (ankylosis, progressive homolog) [37], *TAPT1* (transmembrane anterior posterior transformation 1) [38], and *TRPS1* (trichorhinophalangeal syndrome I) [39] are related to bone metabolism. For *MIR873*, 176 target genes were identified using the TargetScan platform. Several of these genes are also involved in muscle or bone metabolism. For example, *DMD* (dystrophin) and *BMP7* (bone morphogenetic protein 7) are associated with muscle and bone metabolism, respectively [40]. However, the exact mechanisms by which *MIR876* and *MIR873* are involved in co-regulating bone and muscle metabolism are unclear. Functional studies are being planned to validate our findings.

The association results from our initial Chinese sample were replicated in a Caucasian population. Much of the genetic background across the two ethnic groups is similar, which indicates that mutual replication between different ethnic groups is feasible. We found consistent associations across the two ethnic groups for two SNPs (*rs681900* and *rs11859916*), suggesting the consistent effects of these two SNPs on both Chinese and Caucasians. However, ethnic genetic heterogeneity has also been observed among different races when studying specific phenotypes or diseases [41], [42]. Our study found a group of 11 contiguous SNPs ranked at the top for bivariate association with ALM and BR in the Chinese sample, but these findings were not replicated in Caucasians. It is possible that the failure to replicate these findings in Caucasians was attributable to genetic diversity in this region between Chinese and Caucasians.

In conclusion, we used a novel bivariate GWAS approach in a large Chinese sample, and a follow-up replication study in Caucasians, combined with functional evidence, to identify two genes, *HK2* and *UMOD,* that appear to co-regulate FNGPs and ALM. Two additional MicroRNA genes (*MIR873* and *MIR876*) were also associated with bone geometry and ALM in Chinese, but these findings were not replicated in Caucasians. These findings enhance our knowledge of genetic associations between bone geometry and ALM, and provide a rationale for subsequent functional studies of these implicated genes in the pathophysiology of diseases related to these phenotypes, such as hip fracture and sarcopenia.

## Materials and Methods

### Subjects and phenotypes

The study was approved by the institutional review boards of Hunan Normal University, Xian Jiao Tong University and University of Missouri-Kansas City. All study participants signed informed consent documents before they entered the project. Two independent samples were included in this study: a sample of 1,627 unrelated adult Han Chinese (802 males and 825 females) recruited from Changsha and Xi'an and their surroundings areas, and another sample of 2,286 unrelated homogeneous US Caucasians (including 558 males and 1,728 females) recruited from the Midwestern US in Kansas City, Missouri and Omaha, Nebraska. Anthropometric measures and a structured questionnaire including diet, lifestyle, medical history, family information and others were obtained for all subjects.

The five FNGPs, such as W and CSA are calculated based on the BMD (g/cm^2^) and bone size (cm^2^) at the FN. Detailed calculation formulas for the five parameters have been described elsewhere [43], [44], [45]. ALM (g) was calculated as the sum of lean soft tissue (nonfat, non-bone) mass in the arms and legs. BMD and bone size at the FN and ALM were measured by dual-energy X-ray absorptiometry (DXA) with Hologic densitometers (Hologic Inc., Waltham, MA, USA) that were calibrated daily. For Chinese subjects, the coefficient of variation (CV) values of DXA measurements for BMD, bone size at the FN and ALM were 1.87%, 1.94%, and 1.0%, respectively. Similar CV values were obtained with US Caucasians.

### Genotyping

Genomic DNA was extracted from peripheral blood leukocytes using the Puregene DNA isolation kit (Gentra Systems, Minneapolis, MN, USA). All subjects were genotyped using the Human mapping SNP 6.0 assay kit (Affymetrix, Inc, Santa Clara, CA), following the standard protocol recommended by the manufacturer. For quality control (QC) of SNPs, we set the default value of greater than 0.4 as the contrast QC threshold. The final average contrast QC across the entire sample reached the high level of 2.62. In the initial stage, 909,622 SNPs were genotyped for the Chinese subjects. After excluding 17,888 SNP with allele frequencies deviating extremely from Hardy-Weinberg equilibrium (*p*<0.01) and 202,984 SNPs with minor allele frequencies (MAF) <1% (618 SNP were included by both exclusion criteria), a final total of 689,368 SNPs were retained for subsequent analyses, yielding an average marker spacing of ∼4 kb throughout the human genome.

### Statistical Analyses

Although previous studies have reported that FNGPs and body lean mass are two related phenotypes [15], [16], we re-estimated their phenotype correlation used our Chinese data. The bivariate correlation analysis was performed using the statistical package SPSS version 17.0.

We adopted similar statistical analyses in the initial GWAS and replicate study. Before association analyses, raw phenotypes of FNGPs and ALM were adjusted for age and sex. Principal component analysis (PCA) was performed [46] to calculate the principal components, and the ten default main eigenvectors were used as covariates to adjust raw phenotypic data for correction of population stratification.

We performed bivariate GWAS to detect associations between each SNP and two phenotypes. An additive genetic model was applied to both univariate and bivariate association analyses. Based on a linear model, bivariate regression analyses were conducted using the R software package (available at http://www.r-project.org). This method is expressed as follows: for an individual *i*, *y_i_* is a vector of a length of 2 and coding the individual's bivariate phenotype, which can be modeled as
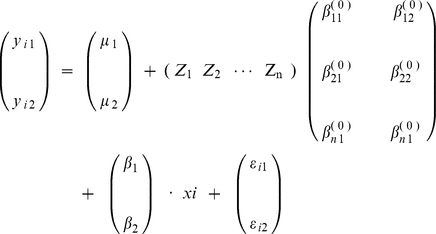



In this model: 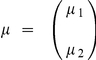 is the grand mean vector; Z = (Z_1_ Z_2_···Z*n*) is a vector coding for covariates, that may include other risk factors and confounding factors; *β's* are the corresponding effects of covariates or the SNP under test; *x_i_* is the genotype score at the locus of interest for individual *i*, and *ε_i_* is the vector of random error. We compared the likelihood of the model under the null hypothesis (SNP effects are restricted to 0), with that under the alternative hypothesis (the SNP effects are not 0), to test the alternative hypothesis. Then the likelihood ratio can convert to an F-statistic, which follows an F-distribution under the null hypothesis. The bivariate *p* value was calculated based on the *F*-statistic.

To compare the results from univariate and bivariate association analyses, we also conducted univariate association with each of the tested phenotypes using Plink (version 1.07, http://pngu.mgh.harvard.edu/~purcell/plink/) in our GWAS and replicate cohorts, where genotypic association analysis was performed under a linear regression framework. Genotype was treated as the independent variable, study phenotype (such as ALM, W) as the dependent variable, and phenotype was modeled as a linear function of alternative genotypes at a certain SNP.

To quantify overall evidence of association achieved in our GWAS and in the US replication cohort, we combined individual p values of the two cohorts using a Fisher's method[47] for meta-analysis. The calculation was performed using the MetaP web tool (http://people.genome.duke.edu/~dg48/metap.php). The linkage disequilibrium (LD) [standardized *D*′(*D*/*D*max)] patterns of interesting SNPs and the haplotype block map was analyzed using Haploview software (available at http://www.broad.mit.edu/mpg/haploview/).

## Supporting Information

Appendix S1 (XLS)
